# Factors That Affect the Accumulation of Strecker Aldehydes in Standardized Wines: The Importance of pH in Oxidation

**DOI:** 10.3390/molecules27103056

**Published:** 2022-05-10

**Authors:** Almudena Marrufo-Curtido, Vicente Ferreira, Ana Escudero

**Affiliations:** Laboratory for Aroma Analysis and Enology, Department of Analytical Chemistry, Faculty of Sciences, Instituto Agroalimentario de Aragón (IA2-Unizar-CITA), Universidad de Zaragoza, 50009 Zaragoza, Spain; amarrufo@unizar.es (A.M.-C.); vferre@unizar.es (V.F.)

**Keywords:** Strecker aldehydes, wine oxidation, pH, oxygen consumption rate, polyphenols

## Abstract

Strecker aldehydes (SA) can be formed in wine from the degradation of Strecker and, to a lesser degree, via the oxidation of higher alcohols. The objective of this article is to assess the magnitude of the differences introduced by wine compositional factors other than amino acids and Fe, in the accumulation of SA during oxidation. Eight red, two rosé and two white wines were oxidized. The accumulation of SA was analyzed. Whites and rosés presented negative accumulations for isobutyraldehyde, and in general, these wines accumulated smaller concentrations of the other SA than red wines. Only methional and phenylacetaldehyde were accumulated in all of the wines during oxidation. 2-methylbutanal and 3-methylbutanal were accumulated in 9 out of the 12 wines, whereas isobutyraldehyde was accumulated only in 5 out of the 12. 2-methylbutanal was, on average, the least accumulated aldehyde. Methional was the aldehyde formed most homogenously. Most of the observed differences can be attributed to three factors: the pH, oxidation time and native levels of Strecker aldehydes. The influence of pH was particularly intense in the cases of phenylacetaldehyde and methional. An independent test using synthetic wines with Strecker amino acids and 4-methylcatechol with different pHs (4.2, 3.5 and 2.8) was carried out in order to verify the higher pH value, the greater accumulation in SA after oxidation process. The results strongly suggest the important role played by pH in the accumulation of SA in wine oxidation.

## 1. Introduction

The post fermentative formation of Strecker aldehydes (SA) in wines could have two origins. The main generation pathway is the Strecker degradation [1], which is a reaction in which the oxidative deamination and decarboxylation of an α-amino acid takes place in the presence of an α-dicarbonyl compound. The result is the formation of an aldehyde that has one carbon less than the original amino acid. Strecker aldehydes include isobutyraldehyde (which comes from the amino acid valine), 3-methylbutanal or isovaleraldehyde, whose precursor is leucine, 2-methylbutanal (isoleucine), phenylacetaldehyde (phenylalanine), and methional (formed from methionine). In principle, any α-dicarbonyl compound can carry out the Strecker aldehyde formation reaction [2]. This includes ortho-quinones formed in the oxidation of ortho-diphenols (reaction confirmed in tea leaves [3]). It has been shown that 4-methylcatechol, having the hydroxyl groups in the ortho position, favors the formation of Strecker aldehydes over polyphenols with the two meta substituents [4]. The models obtained with oxidized wines suggest that the most important compositional factor in the accumulation of SA is the content of precursor amino acids and iron [5]. The second route of formation is the direct oxidation of alcohols. Although its quantitative importance is secondary [5,6], it has been shown that adding higher alcohols to wine or beer increases the concentration of Strecker aldehydes [7,8,9].

Regarding their reactivity, aldehydes are electrophilic species that can be involved in numerous chemical reactions and processes in a reversible and irreversible way. Aldehydes are very reactive with major nucleophilic components of wine such as sulfur dioxide and polyphenols [10,11]. Hydroxyalkylsulfonates are formed with sulfur dioxide [12], which, being non-volatile species, are not perceived, and therefore would not have sensory relevance. The reaction is reversible, and after oxidation of the wine, the balance of the aldehyde–SO_2_ adducts can shift, releasing aldehydes into the environment that are perceived and can change the aromatic profile of the wines [6]. Strecker aldehydes also react with anthocyanin and tannin units to form more stable structures [13,14], following the same mechanism proposed by Timberlake [15] for acetaldehyde-anthocyanin and acetaldehyde-tannin reactions. The reaction product between isobutyraldehyde, 3-methylbutanal and 2-methylbutanal with catechin and malvidin has been characterized both in synthetic samples and in fortified wines from Portugal [14,16,17,18,19], as well as the reaction product between phenylacetaldehyde and malvidin in synthetic wine [13]. Acetals (by reaction with ethanol and glycerol), thioacetals (after reacting with thiols), imines (when found with amino acids) and adducts with glutathione can also be formed [11]. We should take into account all of these interactions to understand the accumulation processes and the study of the reactivity of aldehydes.

It must be noted that to date, some researchers have contributed to improving knowledge on variables that affect Strecker aldehydes’ formation using synthetic wines or solutions [4,20,21,22]. These studies used conditions (temperature, pH) far removed from oenology. There is a lack of research using real wine matrices (red, white and rosé wines) and under oenological conditions.

The main goal of the present paper is to measure the variability introduced in the accumulation of Strecker aldehydes using compositional factors other than the content of precursor amino acids and iron. Matching them in a set of wines will allow us to evaluate the influence of the other parameters in oenological oxidation conditions.

## 2. Results and Discussion

**Scheme 1:**
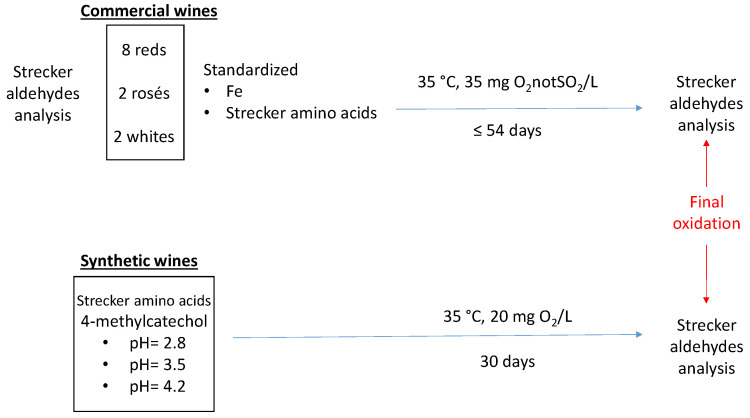
Experiment design and analysis carried out.

Wines (eight reds, two rosés and two whites) used in this experiment and their characteristics appear in Table 1. The wine iron and amino acid content of Strecker was equal in all cases for the wine with the highest concentration of these analytes, in order to cancel these factors and focus the study on the influence of other compositional factors (polyphenols, sulfur dioxide, glycerol, glutathione [11] and others). The objective is not so much to determine the influence of specific factors, but to evaluate the weight in the variability of the accumulation of Strecker aldehydes after oxidation process. See Scheme 1.

Total Strecker aldehydes data before and after oxidation are shown in Table 2. In Table 3, the Strecker aldehyde accumulation rates for the 12 wines are calculated. These rates are calculated by normalizing the accumulation of aldehydes by the oxidation time. It should be noted that the most accumulating wines were also those with the highest pH (3.8) and correspond to the DM-A and MT-Y reds (Table 1, Table 2 and Table 3). The levels of 3-methylbutanal accumulated in the DM-A sample were more than three times higher than those of the next one, and more than an order of magnitude higher than the average of the other six reds. In the case of phenylacetaldehyde, the accumulated levels in DM-A were three times higher than the average of the other three reds. Therefore, as these samples were clearly special, their weight in data processing will be especially taken into account and they will not be considered in this section from now on. The most accumulated aldehyde was phenylacetaldehyde, with a rate of 1.02 μg/L/day in the case of reds (*n* = 6) and 0.44 μg/L/day in whites and rosés. Methional and 3-methylbutanal were the next most accumulated (Table 3). Methional accumulated at 0.55 μg/L/day in reds (*n* = 6) and 0.18 μg/L/day in whites and rosés, and 3-methylbutanal accumulated at 0.54 μg/L/day (*n* = 6) and 0.12 μg/L/day, respectively (Table 3). However, there were important differences between these two aldehydes, since methional clearly accumulated in all of the wines, while 3-methylbutanal did not in two reds and one white.

Isobutyraldehyde and 2-methylbutanal were the least accumulated. Isobutyraldehyde only accumulated (0.21 μg/L/day) in two young red wines (FP-Y and BG-Y), and in whites and rosés, its average accumulation was significantly negative. 2-Methylbutanal did not accumulate in whites and rosés, and in reds it accumulated between 0.07 and 0.30 μg/L/day. Furthermore, it is remarkable that one of the red samples (RB-Y) showed significant negative accumulations of isobutyraldehyde, 2-methylbutanal and 3-methylbutanal.

The lower accumulation rates found in white and rosé wines were not expected according to the hypotheses formulated in previous works [5,23]. Whites and rosés have zero or negligible amounts of anthocyanin [24], which are the molecules suggested as the main consumers of aldehydes [5,25] or sacrificial antioxidants [23]. However, a lower number of phenolic compounds in general could explain a lower amount of Strecker aldehyde precursor quinones. Furthermore, the most important phenolic compounds in white and rosé wines are hydroxycinnamic acids, which can act as free radical scavengers [26]. More studies are needed to elucidate these hypotheses.

It is remarkable that only for reds and without considering DM-A and MT-Y, the variability between wines is low for methional and phenylacetaldehyde (RSD 29% and 55%, respectively). This could indicate that the pH is one of the causes of the differentiation obtained in the wines.

### 2.1. Factors Affecting the Accumulation of Strecker Aldehydes. Discussion of the Main Correlations Found

Correlations are studied between the accumulation of aldehydes expressed either as such or normalized by oxidation time or by O_2_notSO_2_ and various compositional parameters (initial Fe, TPI, pH, initial free SO_2_, initial total SO_2_, final total SO_2_, initial Strecker aldehydes level, initial Strecker’s amino acid level and increase in these amino acids in the experiment). Table S1 in Supplementary Materials shows the correlations found in the 12 wines. Table S2 shows the correlations found with the eight reds. Significant values with p (t) ≤ 0.05 and checked graphically are considered important.

The most relevant correlations found are shown below, regardless of the way of expressing the accumulation rates.

#### 2.1.1. Weight of pH in Variability of SA Accumulation Rate

**Figure 1:**
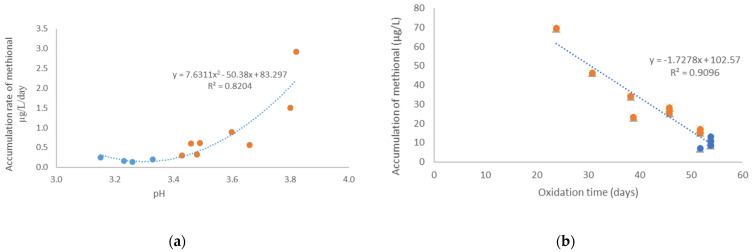
(**a**) Correlation between accumulation rate (µg/L/day) of methional and pH. Blue dots correspond to white and rosé wines. The 8 reds are represented in orange; (**b**) correlation between total accumulation (µg/L) of methional and oxidation time. Blue dots correspond to white and rosé wines. The 8 reds are represented in orange.

A positive correlation with pH for all Strecker aldehydes, more evident for phenylacetaldehyde and methional and more notable for red wines, which showed more diversity of pHs, was observed. A quadratic trend line is shown in Figure 1a.

Since the correlation was essentially motivated by the two wines with the highest pH, the influence of pH on the formation of Strecker aldehydes was verified in a synthetic solution. Model wines containing Strecker amino acids and 4-methylcathecol were tested at wine pH 4.2, 3.5 and 2.8. These results are shown in Table 4. Significantly, as the pH increases, more Strecker aldehyde is accumulated in tested synthetic wine, with the exception of phenylacetaldehyde between pH 4.2 and 3.5. Although in wines the greatest accumulation was observed in samples with pH values of 3.8, in synthetic solutions, significant differences were found in the pH range between 2.8 and 3.5. Monforte et al. observed a greater formation of phenylacetaldehyde in a synthetic medium at 80 °C with phenylalanine at pH 7, compared to pH 3.4 [20]. The greater abundance of amino acids and phenols in deprotonated form -and therefore can react more efficiently in less acidic media has been the hypothesis considered for the greater reactivity of the Strecker reaction.

In addition, in wine there are other causes that can explain the positive correlation between pH and SA accumulation, based on the reactivity of the polyphenolic material and aldehydes. On the one hand, at more basic pHs, the reaction of aldehydes with polyphenols is limited, since it occurs through the carbocation, so the aldehydes will be more stable. In addition, at less acidic pHs, tannins depolymerize less easily, which could explain a lower aldehyde-polyphenol reactivity [27]. On the other hand, at more alkaline pHs, aldehydes are more protected as SO_2_ adducts [24].

#### 2.1.2. Weight of Oxidation Time in Variability of SA Accumulation Rate

A very significant negative correlation between SA formation rate (for any normalization) with oxidation time was observed (Tables S1 and S2). Even the total accumulated amount of methional was correlated with the oxidation time (Figure 1b). This effect is difficult to assign to a single cause, or it is even difficult to establish whether it is a direct or indirect effect. The longer time taken to react SA with polyphenols could explain a direct effect of time. It is remarkable, however, that even the accumulated aldehyde product × reaction time, which should discount this effect, continues to show a highly significant negative correlation with time. This indicates that reaction time is not the only explanation.

Regarding indirect effects, there could be an interaction between the phenolic composition, the O_2_ consumption rate and the SA accumulation. Higher O_2_ consumption rates, related to higher amounts of delphinidin [28], Ref. [23] could be associated with quinones more likely to give the Strecker reaction. Delgado et al. demonstrated under extreme conditions (1 h, 180 °C) that polyphenols with hydroxyl groups in the ortho position (as delphinidin) favored the formation of phenylacetaldehyde [4]. However, reconstituted wines, which rapidly consumed oxygen due to their high levels of highly antioxidant anthocyanins (dephinidin), produced small amounts of Strecker aldehydes [23]. The explanation was that these phenols act as oxygen consumers, but not as precursors of the quinones prone to the Strecker reaction, since they are unstable quinones.

**Figure 2:**
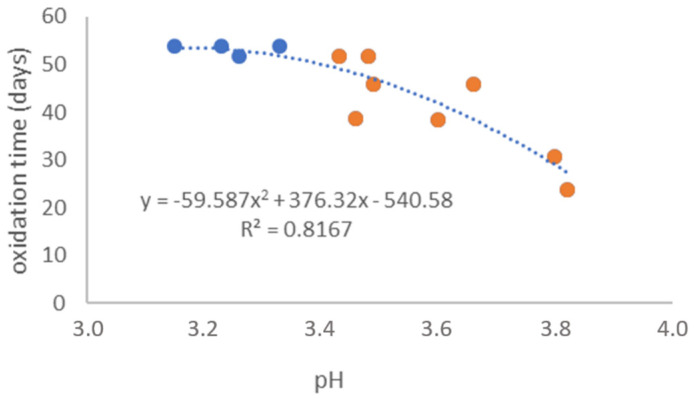
Correlation between oxidation time and pH. Blue dots correspond to white and rosé wines. The 8 reds are represented in orange.

This could also be an effect indirectly associated with pH, since there was a clear positive correlation between the O_2_ consumption rate and pH, as seen in Figure 2. The correlation shown in Figure 2 is lower than the one between aldehyde content and oxidation time. However, in model wines, it was found that (*p* = 0.011) at pH 4.2, oxygen is consumed significantly faster than at pH 3.5, and at the latter pH, faster than at pH 2.8 (Table 4). Oxidation is more favored in more alkaline media, since electron transfer takes place from the phenolate anions [20,29].

#### 2.1.3. Weight of Initial SA Level in Variability of SA Accumulation Rate

**Figure 3:**
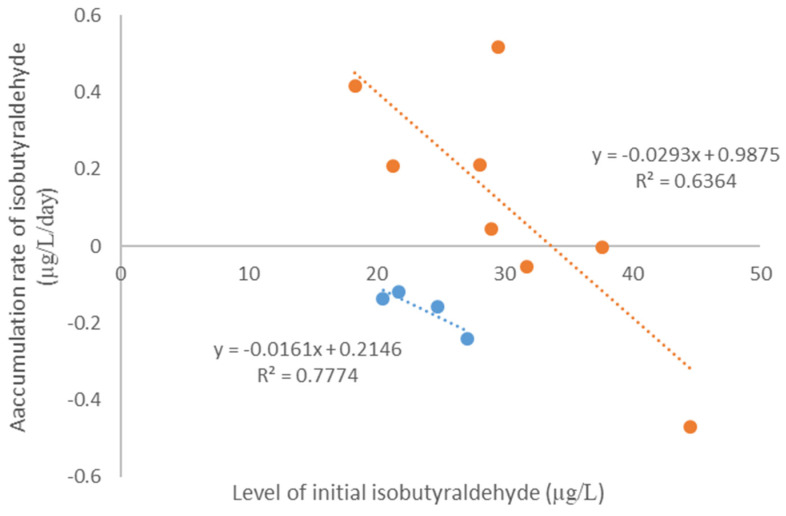
Correlation between isobutyraldehyde accumulation rate (µg/L/day) and initial concentrations of isobutyraldehyde (µg/L). Blue dots correspond to white and rosé wines. The 8 reds are represented in orange.

Negative and significant correlations have been found (for eight red wines) between the accumulation rate of isobutyraldehyde (R = −0.80 *p* = 1.91 × 10^−3^), 3-methylbutanal (R = −0.67 *p* = 1.66 × 10^−2^) (also normalized to O_2_notSO_2_, R = −0.87 *p* = 2.23 × 10^−4^) and 2-methylbutanal (R = −0.77 *p* = 3.17 × 10^−3^) with respect to the initial levels of these aldehydes. The correlation was found in red, white and rosé wines, but considered separately, as seen in Figure 3 for isobutyraldehyde. High levels of aldehydes could be associated with unreactive polyphenolic composition (with respect to the aldehyde-polyphenol reaction), but this would explain a positive correlation, as found for older wines by Bueno et al. [5]. To explain the negative correlation, it could be thought that there was a certain isobutyraldehyde concentration from which this aldehyde became very reactive. This would explain why the three red wines with the highest levels of isobutyraldehyde had negative accumulation coefficients. From these concentrations, the aldehyde-polyphenolic material reactivity would be very high and accelerated by the disappearance of the protective SO_2_ (low isobutyraldehyde- SO_2_ adduct formation constant) [30].

**Figure 4:**
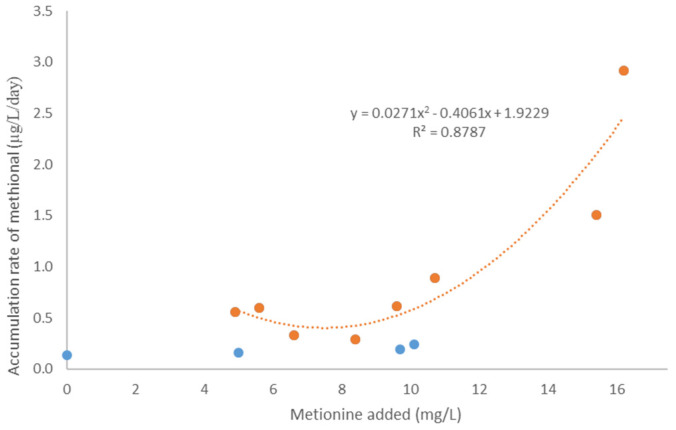
Correlation between methional accumulation rate (µg/L/day) and concentration of added methionine (mg/L). Blue dots correspond to white and rosé wines. The 8 reds are represented in orange.

Additionally, the positive correlations between the accumulation rates of methional and phenylacetaldehyde and the added levels of methionine and phenylalanine (R = 0.75 *p* = 5.09 × 10^−3^ and R = 0.74 *p* = 5.80 × 10^−3^, respectively) were significant (Figure 4 for methional). Considering only the eight red wines, the correlation between the accumulation rate of methional and phenylacetaldehyde per unit of O_2_notSO_2_ and the added levels of methionine and phenylalanine, respectively, improved (R = 0.90 *p* = 6.18 × 10^−5^ and R = 0.96 *p* = 5.66 × 10^−7^, respectively). This could suggest that the added amino acid was more reactive than that present or native in the samples, perhaps because the native amino acids form some slow and reversible interactions with other wine components and therefore they have lower reactivity than added amino acids. The native aldehydes could be the components with which the amino acids could establish interactions. The causality found should be studied, since according to the bibliographic study, there is no clear evidence that amino acids can form strong interactions. In addition, the correlation was highly mediated by the two wines with higher formation rates (higher pH). If these two wines were excluded, the correlation would disappear completely (Figure 4).

## 3. Materials and Methods

### 3.1. Solvents and Chemicals

Sodium hydrogencarbonate, sulphuric acid (96%), ortho phosphoric acid (85%), hydrogen peroxide 3% stabilized *w*/*v* VINIKIT, indicator 4,4, mixed (methyl red-methylene blue) VINIKIT, and sodium hydroxide 0.01 molL^−1^ VINIKIT were from Panreac (Barcelona, Spain). L(+)-tartaric acid (99%), glycerol (99.5%), iron (II) chloride tetrahydrate (>99%), manganese (II) choride tetrahydrate (>99%), copper (I) chloride (99.9%), L-leucine (Leu) (>98%), L-isoleucine (Ile) (>98%), D-valine (Val) (>98%), L-phenylalanine (Phe) (>98%), D-methionine (Met) (>98%) and 4-methylcatechol (4m-catol) (>95%) were from Sigma-Aldrich Madrid, Spain. Isolute ENV+sorbent, 1 mL cartridges, PTFE frits and ethanol were purchased from Merck (Darmstadt, Germany). Sodium hydroxide 99% was purchased from Scharlab (Sentmenat, Spain). Isobutyraldehyde (99%), 2-methylbutanal (95%), phenylacetaldehyde (95%) and methional (98%), 2-methylpentanal (98%), 3-methylpentanal (97%) and O-(2,3,4,5,6-pentafluorobenzyl)hydroxylamine hydrochloride (PFBHA 98%) were supplied by Merck USA. Phenylacetaldehyde-d2 (95%) and methional-d2 were purchased from Eptes (Vevey, Switzerland). Water was purified in a Milli-Q system from Millipore (Bedford, Germany).

### 3.2. Samples and Oxidation Procedure

#### 3.2.1. Commercial Wines

Wines used in this experiment were all commercial, and their characteristics appear in Table 1. The pH value was determined by the potentiometric method [31], the free and total SO_2_ was determined by the Rankine method following the recommendations of the OIV [32], and the total polyphenol index (TPI) was determine by spectrophotometric measurement at 280 nm as described by Ribéreau-Gayon et al. [31]. Iron was also determined by ICP-MS following the procedure described by Grindlay et al. [33]. The determination of valine, methionine, isoleucine, leucine and phenylalanine was carried out according to the method reported by Hernández–Orte et al. [34].

Afterwards, each wine was introduced into the oxygen-free chamber where its iron and Strecker amino acids levels were corrected, adding the necessary amount to bring them in all cases to the maximum level found in the set of samples Table 1. The maximum values were 2.16 mgL^−1^ of Fe, 68.9 mgL^−1^ of valine, 51.2 mgL^−1^ of isoleucine, 108.4 mgL^−1^ of leucine, 26.4 mgL^−1^ of methionine and 70.4 mgL^−1^ of phenylalanine. Wines were removed one by one and filtered through amicrobic filters of 73 mm diameter and 0.22 µm pore size (MERK, REF: SCGP U02 RE, Damrstadt, Germany)). Once filtered, wines were saturated with air and placed in screw-cap vials of 60 mL with the headspace adjusted to the volume strictly necessary to contain the level of O_2_ required to oxidize all of the SO_2_ contained in that wine plus 35 mgL^−1^, as described in Marrufo-Curtido et al. [35]. The stoichiometrically required amount of oxygen to oxidize the SO_2_ was calculated from total SO_2_ after oxidation, as shown in Table 2, and hence the O_2_notSO_2_ that would have been invested in producing Strecker aldehydes. For each wine, two independent vials were prepared with SPT3 sensors (Nomacorc SA, Thimister-Clermont, Belgium) and incubated at 35 °C in a water bath with orbital shaking at 90 rpm “Grant OLS23” (Grant Instruments Ltd., Cambridge, UK). Dissolved oxygen levels were measured twice a day for the first week and then once a day until the end of oxidation (at which point each tube had consumed 95% of the available oxygen or after a maximum of 54 days of oxidation).

Once the oxidation was finished, for each vial, the total Strecker aldehydes were analyzed [36] and total SO_2_ by Rankine [32].

#### 3.2.2. Synthetic Wines

Water containing 5 g/L of tartaric acid was spiked with glycerol (5 g/L), FeCl_2_·4 H_2_O, (5 mg/L Fe), MnCl_2_·4 H_2_O (0.2 mg/L Mn) and CuCl (0.2 mg/L Cu) to form three synthetic wines containing 12% (*v*/*v*) ethanol [21,37]. After this, synthetic wines were spiked with 1 mM of Leu, 1 mM of Ile, 1 mM of Val, 1 mM of Phe, 1 mM of Met and 1 mM of 4m-catol. The first synthetic wine was adjusted to pH 4.2, the second one was adjusted to pH 3.5, and the last one was adjusted to pH 2.8. Two blanks adjusted to pH 3.5 were prepared, one with only amino acids and the other with only 4m-catol. Synthetic wines and blanks were saturated with air and placed in vials of 12 mL with the headspace adjusted to the volume strictly necessary to contain 20 mgL^−1^ of O_2_ according to the method reported by Marrufo-Curtido et al. [35]. For each synthetic wine and blanks, two independent vials were prepared with SPT3 sensors (Nomacorc SA, Thimister-Clermont, Belgium) and incubated at 35 °C, with agitation. Dissolved oxygen levels were measured once a day, until the end of oxidation (30 days of oxidation). Once the oxidation was finished, for each vial the total Strecker aldehydes were analyzed [36] and the consumed O_2_ rate calculated.

### 3.3. Strecker Aldehydes Determination

The determination of total Strecker aldehydes (isobutyraldehyde, 2-methylbutanal, 3-metylbutanal, methional and phenylacetaldehyde) in wine is described in the method proposed by Castejón-Musulén et al. [36]. This method is based on analyzing SO_2_-bonded aldehydes in wine using O-(2,3,4,5,6-pentafluorobenzyl) hydroxylamine (PFBHA), obtaining the corresponding oximes of each analyte, which will be extracted by solid phase extraction (SPE) and further quantified by gas chromatography coupled to mass spectrometry.

#### 3.3.1. Chromatographic System

A Shimadzu GC-2010 chromatograph was used, with a SPL1 injector splitless mode with a DB-WAX ETR column (30 m × 0.25 mm × 0.5 μm). As a detector, a Shimadzu GCMS-QP 2010 quadrupole was used, and as automatic injector, the Shimadzu AOC-5000 was selected. The quantification ion for isobutyraldehyde was 250 (*m*/*z*), that for 2-methylbutanal was 239 (*m*/*z*), for 3-metylbutanal it was 239 (*m*/*z*), for methional it was 299 (*m*/*z*) and for phenylacetaldehyde it was 297 (*m*/*z*).

#### 3.3.2. Chromatographic Conditions

The flow through the column was 1.26 mL/min, with a linear velocity of 40.5 cm/s and a purge of 1.0 mL/min, using helium as carrier gas. The temperature program was 40 °C for 4 min followed by a ramp of 10 °C/min until reaching 250 °C, which is maintained for 5 min. Three microliters of the sample was injected using a pressure pulse of 300 kPa. The injector was kept at 250 °C for 1.5 min.

#### 3.3.3. Experimental Procedure

Twelve milliliters of a wine sample spiked with 30 μL of standard internal dissolution: deuterated methional, deuterated phenylacetaldehyde, 2-methylpentanal and 3-methylpentanal (50 μg/L) was transferred to a vial inside an oxygen free chamber from Jacomex (Dagneux, France). Then, the sample was incubated at 50 °C for 6 h in order to equilibrate the matrix, thereby ensuring that all the adducts (SO_2_-aldehyde) and (polyphenol-aldehyde) can be established. The next step consisted of adding 360 μL of PFBHA (0.3 g/L) as the derivatized agent and incubated for 12 h at 35 °C. After this time, the vials were removed from the oven. For each vial, a 1 mL capacity cartridge containing 30 mg of LiChrolut^®^ was prepared. Ten milliliters of the sample was added to the cartridge, then 10 mL of the wash solution was added (water/methanol solution at 60 (*v*/*v*) in methanol and 1% (m/m) in sodium hydrogen carbonate). Finally, each cartridge previously dried was eluted with 1.2 mL of hexane. The quantification method was the response factor obtained with known concentrations in the same matrices

### 3.4. Data Treatment and Statistical Analysis

Correlation studies and one-factor analysis of variance (ANOVA) were carried out with Excel 2016 (Microsoft, Washington, DC, USA). For significant effects, Fischer post hoc pairwise comparison (95%) tests were performed.

## 4. Conclusions

Remarkably, most of the observed differences can be attributed to three factors: the pH, the time required by each wine to consume all of the O_2_ delivered (oxidation time) and native levels of Strecker aldehydes. Initial levels of Strecker aldehydes could be associated with polyphenol profile. The influence of pH was particularly intense in the cases of phenylacetaldehyde and methional, while the oxidation time was more important for isobutyraldehyde and 2 and 3-methylbutanals. An independent test using synthetic wines was carried out in order to verify the higher pH value, the greater accumulation in Strecker aldehydes. Given that pH and oxidation time are both related to the stability of the aldehydes in the medium and that pH is also likely associated with the Strecker degradation kinetics, the results strongly suggest that the role played by pH in the accumulation of aldehydes is very important.

## Figures and Tables

**Table 1:** Characteristics of the samples and some initial compositional parameters. Fe, Strecker amino acids, free SO_2_ and Total SO_2_ are expressed in mg/L.

Type of Wine	Code	Variety	Vintage	Fe	Valine	Isoleucine	Luecine	Methionine	Phenylalanine	pH	TPI ^7^	Free SO_2_	Total SO_2_
	CV-A ^1^	G/T/Cr ^5^	2008	1.3	44.6	33.9	95.3	16.8	58.8	3.49	53.0	4.8	62.8
	DM-A	Tempranillo	2012	1.2	38.7	31.4	41.1	10.1	34.1	3.82	55.5	3.2	20.0
	CL-A	Tempranillo	2013	1.9	68.9	48.4	105.0	21.5	69.0	3.66	46.3	11.2	81.0
Red wines	VF-A	Garnacha	2015	1.2	48.5	36.0	68.8	19.7	59.9	3.48	54.7	11.2	124.0
	MT-Y ^2^	Tempranillo	2018	0.9	30.2	22.8	33.1	11.0	33.3	3.80	61.7	8.8	57.0
	FP-Y	Tempranillo	2018	1.8	42.2	29.5	52.0	15.7	46.7	3.60	46.7	12.8	52.0
	BG-Y	Garnacha	2018	2.2	52.3	33.7	70.9	20.8	63.1	3.46	50.5	11.2	37.0
	RB-Y	Garnacha	2018	1.2	41.6	29.7	59.2	18.0	60.9	3.43	44.1	3.2	96.0
Rosé	VMG-R ^3^	Garnacha	2018	0.4	60.6	51.2	108.0	26.4	70.4	3.26	11.7	3.84	86.0
wines	GF-R	G/T/M/C/Sy ^6^	2018	2.1	30.6	25.1	64.1	16.2	47.4	3.15	13.3	5.6	78.0
White	VT-W ^4^	Viura	2018	0.6	52.7	33.1	87.2	21.4	64.9	3.23	10.7	14.4	115.0
wines	VM-W	Viura	2018	0.2	41.3	28.0	65.0	16.7	41.6	3.33	7.41	10.4	97.0

^1^ A represents aged red wines and ^2^ Y means young red wines. ^3^ R means rosé wine. ^4^ W means white wine. ^5^ G/T/Cr: Grenache-Tempranillo-Cariñena ^6^ G/T/M/C/Sy: Grenache-Tempranillo-Merlot-Cabernet Sauvignon-Syrah. ^7^ TPI (total polyphenol index).

**Table 2:** Parameters related with the oxidation experiment and levels of total Strecker aldehydes (μg/L) initial and after oxidation process. s: standard deviation (*n* = 2).

Code	Total SO_2_ (mg/L)	O_2_notSO_2_^5^ (mg/L)	s	Days of Oxidation	Isobutyraldehyde	2-methylbutanal	3-methylbutanal	Methional	Phenylacetaldehyde
CV-A ^1^ initial	62.8	-	-		37.6 ± 1.8	8.32 ± 0.4	27.4 ± 1.0	3.1 ± 0.0	19.4 ± 0.6
CV-A after oxidation	19.2	27.57	0.8	45.8	37.5 ± 0.6	15.4 ± 0.0 *	52.0 ± 1.1 *	31.2 ± 0.6 *	59.4 ± 1.6 *
DM-A initial	20.0	-	-		29.5 ± 1.2	7.0 ± 0.2	15.7 ± 1.1	1.8 ± 0.3	12.6 ± 1.1
DM-A after oxidation	11.2	34.48	0.2	23.8	41.8 ± 1.0 *	17.9 ± 0.2 *	178.1 ± 1.0 *	71.3 ± 0.2 *	188.5 ± 0.9 *
CL-A initial	81.0	-	-		31.7 ± 0.3	5.08 ± 0.1	69.1 ± 0.9	2.7 ± 0.0	30.7 ± 0.2
CL-A after oxidation	37.6	38.47	0.4	45.8	29.3 ± 0.4 *	9.93 ± 0.3 *	55.0 ± 8.3	28.1 ± 0.4 *	63.1 ± 0.2 *
VF-A initial	124	-	-		29.0 ± 14	6.79 ± 3.3	43.7 ± 21.8	4.9 ± 2.2	43.6 ± 19.7
VF-A after oxidation	15.2	22.6	0.8	51.8	31.4 ± 0.7	10.6 ± 0.0	79.3 ± 1.0	21.8 ± 0.3 *	67.0 ± 0.6
MT-Y ^2^ initial	57.0	-	-		18.3 ± 1.7	4.3 ± 0.7	26.9 ± 4.7	1.4 ± 0.4	12.9 ± 1.1
MT-Y after oxidation	19.4	33.71	0.4	30.8	31.1 ± 1.2 *	19.1 ± 0.4 *	93.8 ± 3.3 *	47.7 ± 0.2 *	155.0 ± 0.8 *
FP-Y initial	52.0	-	-		28.1 ± 1.2	5.24 ± 0.2	38.8 ± 1.2	2.4 ± 0.0	22.3 ± 0.5
FP-Y after oxidation	25.6	36.5	0.6	38.3	36.2 ± 2.5	16.8 ± 1.1 *	124.6 ± 10.3 *	36.5 ± 1.1 *	112.0 ± 4.3 *
BG-Y initial	37.0	-	-		21.3 ± 0.3	4.13 ± 0.1	10.3 ± 0.2	1.4 ± 0.0	11.0 ± 0.0
BG-Y after oxidation	21.6	36.12	0.4	38.8	29.4 ± 1.0 *	14.8 ± 0.4 *	69.9 ± 0.5 *	24.5 ± 0.6 *	59.7 ± 0.6 *
RB-Y initial	96.0	-	-		44.5 ± 4.6	19.9 ± 2.2	129.3 ± 15.2	2.9 ± 0.0	17.6 ± 0.3
RB-Y after oxidation	26.9	26.26	0.1	51.8	20.2 ± 0.0 *	9.01 ± 0.1 *	53.1 ± 1.8 *	18.1 ± 0.2 *	43.6 ± 0.9 *
VMG-R ^3^ initial	86.0	-	-		21.7 ± 0.4	6.56 ± 0.0	21.4 ± 0.0	1.4 ± 0.0	8.7 ± 0.3
VMG-R after oxidation	40.0	23.65	0.8	51.8	15.6 ± 0.9 *	5.45 ± 0.2 *	40.2 ± 0.6 *	8.4 ± 0.0 *	28.0 ± 0.4 *
GF-R initial	78.0	-	-		24.8 ± 0.5	4.18 ± 0.2	22.8 ± 0.1	1.7 ± 0.0	7.8 ± 0.3
GF-R after oxidation	31.5	21.51	0.8	53.8	16.4 ± 0.5 *	5.97 ± 0.2 *	47.2 ± 3.5 *	14.7 ± 0.4 *	45.7 ± 1.2 *
VT-W ^4^ initial	115	-	-		20.5 ± 1.1	4.79 ± 0.1	44.6 ± 3.4	10.9 ± 0.0	18.3 ± 0.3
VT-W after oxidation	60.8	13.68	2.5	53.8	13.1 ± 0.4 *	4.93 ± 0.1	55.3 ± 1.5	19.5 ± 0.1 *	29.6 ± 0.3 *
VM-W initial	97.0	-	-		27.1 ± 3.7	9.53 ± 1.3	70.5 ± 5.4	2.1 ± 0.0	6.9 ± 0.2
VM-W after oxidation	33.1	16.94	1.3	53.8	14.1 ± 0.5	4.97 ± 0.2	41.8 ± 1.9 *	12.6 ± 0.3 *	32.4 ± 0.4 *

^1^ A represents Aged red wines and ^2^ Y means Young red wines. ^3^ R means Rosé wine. ^4^ W means White wine. ^5^ O_2_notSO_2_ means the amount of O_2_ consumed not invested in the oxidation of wine SO_2_. * represents significant differences between initial and after oxidation (*p* < 0.05) (Fisher post hoc test).

**Table 3:** Accumulation rates (µg/L/day) of Strecker aldehydes in the 12 wines after the oxidation process, considering the days of oxidation (23.9–51.8 days). Average, deviation and RSD (%) of the whole group and of reds, reds without the wines that have pH 3.8 and W and R wines: whites and rosés. The results of the analysis of variance ANOVA (*p* = 0.05) are shown in capital letters. Different letters indicate significant differences.

	Isobutyraldehyde	2-methylbutanal	3-methylbutanal	Methional	Phenylacetaldehyde
CV-A ^1^	−2.14 × 10^−3^	0.15	0.55	0.62	0.88
DM-A	0.41	0.37	5.45	2.33	5.90
CL-A	−0.04	0.08	−0.22	0.40	0.51
VF-A	0.06	0.07	0.84	0.40	0.55
MT-Y ^2^	0.34	0.39	1.78	1.23	3.77
FP-Y	0.21	0.30	1.67	0.66	1.75
BG-Y	0.21	0.21	1.17	0.46	0.96
RB-Y	−0.49	−0.22	−1.54	0.31	0.53
VMG-R ^3^	−0.24	−0.04	0.73	0.27	0.75
GF-R	−0.25	0.05	0.74	0.4	1.15
VT-W ^4^	−0.16	0	0.23	0.19	0.25
VM-W	−0.31	−0.11	−0.69	0.25	0.61
Total average	−0.03 **C**	0.10 **C**	0.89 **AB**	0.63 **BC**	1.47 **A**
s	0.27	0.18	1.72	0.6	1.68
RSD (%)	-	>100	>100	97	>100
Average red wines	0.07 **C**	0.16 **C**	1.21 **AB**	0.80 **BC**	1.86 **A**
s	0.28	0.19	2.03	0.68	1.97
RSD (%)	>100	>100	>100	85	>100
Average red wines except DM-A and MT-Y	−9.70 × 10^−3^ **B**	0.09 **B**	0.54 **AB**	0.55 **AB**	1.02 **A**
s	0.24	0.16	1.15	0.14	0.47
RSD (%)	-	>100	>100	29	55
Average W and R wines	−0.24 **C**	−0.02 **BC**	0.12 **AB**	0.18 **AB**	0.44 **A**
s	0.06	0.07	0.67	0.09	0.37
RSD (%)	-	-	>100	32	54

^1^ A represents Aged red wines and ^2^ Y means Young red wines. ^3^ R means Rosé wine. ^4^ W means White wine.

**Table 4:** Averages and standard deviation (*n* = 2) of consumed oxygen rate (mg/L/day) and Strecker aldehydes concentrations (μg/L). Different letters indicate significant differences (*p* < 0.05) (Fisher post hoc test).

	pH 4.2	pH 3.5	pH 2.8	*p*
Cons. O_2_ rate (mg /L/day)	0.68 ± 0.02 ^a^	0.60 ± 0.01 ^b^	0.53 ± 0.02 ^c^	0.011
Isobutyraldehyde	1364.2 ± 4.8 ^a^	1109.9 ± 61.9 ^b^	886.0 ± 52.3 ^c^	0.005
2-methylbutanal	1476.8 ± 35.2 ^a^	1206.3 ± 34.9 ^b^	976.2 ± 63.5 ^c^	0.004
3-methylbutanal	2833.6 ± 0.1 ^a^	2009.5 ± 132.0 ^b^	1217.1 ± 122.8 ^c^	0.001
Methional	4286.6 ± 150.3 ^a^	3575.4 ± 50.4 ^b^	3043.3 ± 241.4 ^c^	0.011
Phenylacetaldehyde	3026.7 ± 78.9 ^a,b^	3230.0 ± 3.3 ^a^	2845.8 ± 180.0 ^b^	0.094

## Data Availability

Not applicable.

## Supplementary Materials

The following supporting information can be downloaded at https://www.mdpi.com/article/10.3390/molecules27103056/s1: Table S1: Correlations between all the variables and the different ways of expressing the accumulation for all the wines. Significant values with p (t) ≤ 0.05 highlighted in bold and checked graphically. Table S2: Correlations between all the variables and the different ways of expressing the accumulation for red wines. Significant values with p (t) ≤ 0.05 highlighted in bold and checked graphically.
