# Supplementary material for: Factors That Affect the Accumulation of Strecker Aldehydes in Standardized Wines: The Importance of pH in Oxidation
Source: Molecules. 2022 May 10;27(10):3056. doi: 10.3390/molecules27103056 (PMC9146978; doi:10.3390/molecules27103056)
Supplement: Supplementary file 1 [file molecules-27-03056-s001.zip › molecules-1704117-supplementary.pdf]

Supplementary Materials:

Table S1. Correlations between all the variables and the different ways of expressing the accumulation for all the wines. Significant values with p (t) ≤ 0.05 highlighted in bold and checked graphically

			**Accumulation = final - initial (µg/L)**					**Accumulation/ time (µg/L / days)**					**Accumulation/ O_2 no SO_2 ((µg/L)/ mg/L)**				
			**Isobutyraldehyde**	**2-methylbutanal**	**3-methylbutanal**	**Methional**	**Phenylacetaldehyde**	**Isobutyraldehyde**	**2-methylbutanal**	**3-methylbutanal**	**Methional**	**Phenylacetaldehyde**	**Isobutyraldehyde**	**2-methylbutanal**	**3-methylbutanal**	**methional**	**Fenilacetaldehído**
	Initial Fe	R	**0.26**	**0.38**	**0.20**	**0.20**	**0.10**	0.20	0.28	0.12	0.09	0.04	0.37	0.40	0.22	0.05	0.01
		p(t)	**4.08E-01**	**2.23E-01**	**5.32E-01**	**5.39E-01**	**7.51E-01**	5.37E-01	3.83E-01	7.08E-01	7.76E-01	9.07E-01	2.43E-01	1.93E-01	4.88E-01	8.73E-01	9.66E-01
	Added Fe	R	**-0.26**	**-0.38**	**-0.20**	**-0.20**	**-0.10**	-0.20	-0.28	-0.12	-0.09	-0.04	-0.37	-0.40	-0.22	-0.05	-0.01
		p(t)	**4.08E-01**	**2.23E-01**	**5.32E-01**	**5.39E-01**	**7.51E-01**	5.37E-01	3.83E-01	7.08E-01	7.76E-01	9.07E-01	2.43E-01	1.93E-01	4.88E-01	8.73E-01	9.66E-01
	PTI	R	**0.61**	**0.61**	**0.42**	**0.69**	**0.57**	**0.61**	**0.64**	0.44	**0.57**	0.52	**0.70**	**0.60**	0.39	**0.60**	0.46
		p(t)	**3.65E-02**	**3.37E-02**	**1.69E-01**	**1.26E-02**	**5.51E-02**	**3.47E-02**	**2.42E-02**	1.57E-01	**5.28E-02**	8.37E-02	**1.05E-02**	**3.71E-02**	2.11E-01	**3.90E-02**	1.31E-01
	pH	R	**0.67**	**0.66**	**0.55**	**0.88**	**0.80**	**0.74**	**0.76**	**0.63**	**0.80**	**0.77**	**0.70**	**0.59**	**0.46**	**0.78**	**0.71**
		p(t)	**1.64E-02**	**2.04E-02**	**6.64E-02**	**1.70E-04**	**1.98E-03**	**5.89E-03**	**3.89E-03**	**2.96E-02**	**1.79E-03**	**3.43E-03**	**1.06E-02**	**4.39E-02**	1.31E-01	**2.55E-03**	**9.91E-03**
	Time of oxidation	R	**-0.81**	**-0.79**	**-0.81**	**-0.95**	**-0.94**	**-0.90**	**-0.92**	**-0.87**	**-0.93**	**-0.93**	**-0.79**	**-0.72**	**-0.72**	**-0.87**	**-0.88**
		p(t)	**1.41E-03**	**2.17E-03**	**1.32E-03**	**1.47E-06**	**3.91E-06**	**5.28E-05**	**2.15E-05**	**2.56E-04**	**1.43E-05**	**1.47E-05**	**2.37E-03**	**8.40E-03**	**7.98E-03**	**2.31E-04**	**1.66E-04**
	Total consumed O_2	R	**0.24**	**0.24**	**-0.02**	**0.23**	**0.11**	0.18	0.19	-0.04	0.10	0.05	0.40	0.24	-0.03	0.08	0.00
		p(t)	**4.45E-01**	**4.52E-01**	**9.41E-01**	**4.70E-01**	**7.26E-01**	5.73E-01	5.46E-01	8.96E-01	7.63E-01	8.74E-01	1.96E-01	4.53E-01	9.26E-01	7.97E-01	9.97E-01
	O_2 no SO_2	R	**0.61**	**0.66**	**0.46**	**0.66**	**0.58**	**0.61**	**0.67**	0.45	0.55	0.51	**0.70**	**0.60**	0.39	0.44	0.43
		p(t)	**3.41E-02**	**1.98E-02**	**1.36E-01**	**1.93E-02**	**4.99E-02**	**3.54E-02**	**1.71E-02**	1.37E-01	6.44E-02	9.00E-02	**1.09E-02**	**4.12E-02**	2.12E-01	1.53E-01	1.62E-01
	Initial	R	0.22	0.24	-0.02	-0.22	-0.21	0.09	0.09	-0.17	-0.28	-0.27	0.13	0.21	0.01	-0.23	-0.25
	Free SO_2	*p*(t)	4.82E-01	4.48E-01	9.60E-01	4.96E-01	5.04E-01	7.72E-01	7.74E-01	5.88E-01	3.75E-01	3.97E-01	6.92E-01	5.07E-01	9.71E-01	4.77E-01	4.38E-01
	Initial	R	**-0.65**	**-0.69**	**-0.72**	**-0.78**	**-0.77**	**-0.73**	**-0.77**	**-0.74**	**-0.75**	**-0.74**	**-0.65**	**-0.61**	**-0.61**	**-0.65**	**-0.70**
	Total SO_2	*p*(t)	**2.08E-02**	**1.29E-02**	**8.88E-03**	**2.98E-03**	**3.62E-03**	**7.61E-03**	**3.19E-03**	**5.54E-03**	**5.16E-03**	**6.30E-03**	**2.33E-02**	**3.38E-02**	**3.34E-02**	**2.11E-02**	**1.09E-02**
	Final total SO_2	R	-0.50	-0.46	-0.48	**-0.64**	**-0.58**	-0.54	-0.53	-0.51	**-0.58**	-0.55	**-0.58**	-0.44	-0.40	**-0.60**	-0.56
		p(t)	9.93E-02	1.31E-01	1.14E-01	**2.53E-02**	**4.57E-02**	6.85E-02	7.57E-02	8.78E-02	**5.02E-02**	6.30E-02	**4.93E-02**	1.53E-01	1.95E-01	**3.95E-02**	5.87E-02
Level of native aldehyde	Isobutyraldehyde	R	-0.47	-0.44	-0.40	0.02	-0.16	-0.43	-0.39	-0.25	-0.04	-0.14	-0.35	-0.43	-0.45	0.02	-0.19
		p(t)	1.20E-01	1.52E-01	1.96E-01	9.58E-01	6.13E-01	1.64E-01	2.06E-01	4.42E-01	9.05E-01	6.66E-01	2.69E-01	1.65E-01	1.41E-01	9.60E-01	5.52E-01
	2-methylbutanal	R	**-0.69**	**-0.71**	**-0.57**	-0.17	-0.22	**-0.59**	**-0.58**	-0.37	-0.15	-0.18	**-0.63**	**-0.74**	**-0.66**	-0.14	-0.20
		p(t)	**1.22E-02**	**9.35E-03**	**5.07E-02**	5.93E-01	5.00E-01	**4.37E-02**	**4.87E-02**	2.37E-01	6.37E-01	5.80E-01	**2.87E-02**	**6.09E-03**	**2.03E-02**	6.67E-01	5.28E-01
	3-methylbutanal	R	**-0.76**	**-0.74**	**-0.77**	-0.33	-0.38	**-0.70**	**-0.66**	**-0.60**	-0.35	-0.37	**-0.71**	**-0.77**	**-0.83**	-0.33	-0.38
		p(t)	**4.43E-03**	**6.44E-03**	**3.50E-03**	2.88E-01	2.23E-01	**1.12E-02**	**1.87E-02**	**3.98E-02**	2.65E-01	2.38E-01	**9.78E-03**	**3.16E-03**	**9.24E-04**	2.98E-01	2.22E-01

	Methional	R	-0.21	-0.23	-0.19	-0.32	-0.37	-0.25	-0.28	-0.22	-0.28	-0.31	-0.29	-0.16	-0.08	-0.17	-0.34
		p(t)	5.07E-01	4.71E-01	5.62E-01	3.06E-01	2.32E-01	4.36E-01	3.72E-01	5.01E-01	3.70E-01	3.26E-01	3.57E-01	6.29E-01	7.94E-01	6.01E-01	2.75E-01
	Phenylacetaldehyde	R	0.13	0.09	-0.06	-0.02	-0.19	0.02	-0.03	-0.13	-0.11	-0.19	0.23	0.16	0.02	-0.03	-0.26
		p(t)	6.94E-01	7.91E-01	8.48E-01	9.52E-01	5.53E-01	9.50E-01	9.36E-01	6.92E-01	7.30E-01	5.49E-01	4.66E-01	6.12E-01	9.47E-01	9.29E-01	4.11E-01
Level of native aminoacids	Valine	R	-0.12	-0.17	-0.27	-0.35	-0.50	-0.23	-0.28	-0.31	-0.35	-0.44	-0.05	-0.14	-0.21	-0.47	**-0.61**
		p(t)	7.13E-01	6.07E-01	3.97E-01	2.62E-01	9.72E-02	4.76E-01	3.75E-01	3.33E-01	2.64E-01	1.52E-01	8.66E-01	6.60E-01	5.19E-01	1.25E-01	**3.65E-02**
	Isoleucine	R	-0.09	-0.16	-0.17	-0.27	-0.40	-0.18	-0.25	-0.20	-0.25	-0.33	0.01	-0.12	-0.10	-0.39	-0.49
		p(t)	7.74E-01	6.14E-01	5.97E-01	3.88E-01	2.00E-01	5.79E-01	4.34E-01	5.39E-01	4.30E-01	2.95E-01	9.82E-01	7.16E-01	7.62E-01	2.06E-01	1.07E-01
	Leucine	R	-0.35	-0.34	-0.45	**-0.60**	**-0.73**	-0.49	-0.51	-0.52	**-0.60**	**-0.68**	-0.28	-0.26	-0.34	**-0.63**	**-0.77**
		p(t)	2.59E-01	2.77E-01	1.44E-01	**3.98E-02**	**7.28E-03**	1.10E-01	9.25E-02	8.00E-02	**4.04E-02**	**1.56E-02**	3.73E-01	4.07E-01	2.81E-01	**2.69E-02**	**3.56E-03**
	Methionine	R	-0.42	-0.46	-0.51	**-0.78**	**-0.82**	**-0.56**	**-0.61**	**-0.61**	**-0.75**	**-0.78**	-0.36	-0.40	-0.41	**-0.84**	**-0.87**
		p(t)	1.72E-01	1.35E-01	8.76E-02	**2.83E-03**	**1.11E-03**	**5.98E-02**	**3.44E-02**	**3.36E-02**	**5.09E-03**	**2.73E-03**	2.47E-01	2.02E-01	1.82E-01	**5.56E-04**	**2.42E-04**
	Phenylalanine	R	-0.41	-0.41	-0.52	**-0.66**	**-0.78**	-0.54	-0.56	**-0.60**	**-0.67**	**-0.74**	-0.30	-0.33	-0.41	**-0.73**	**-0.86**
		p(t)	1.89E-01	1.89E-01	7.98E-02	**2.03E-02**	**2.64E-03**	7.00E-02	5.58E-02	**3.93E-02**	**1.80E-02**	**5.80E-03**	3.38E-01	3.02E-01	1.81E-01	**7.05E-03**	**3.01E-04**
Aminoacid increase	Valine	R	0.12	0.17	0.27	0.35	0.50	0.23	0.28	0.31	0.35	0.44	0.05	0.14	0.21	0.47	**0.61**
		p(t)	7.13E-01	6.07E-01	3.97E-01	2.62E-01	9.72E-02	4.76E-01	3.75E-01	3.33E-01	2.64E-01	1.52E-01	8.66E-01	6.60E-01	5.19E-01	1.25E-01	**3.65E-02**
	Isoleucine	R	0.09	0.16	0.17	0.27	0.40	0.18	0.25	0.20	0.25	0.33	-0.01	0.12	0.10	0.39	0.49
		p(t)	7.74E-01	6.14E-01	5.97E-01	3.88E-01	2.00E-01	5.79E-01	4.34E-01	5.39E-01	4.30E-01	2.95E-01	9.82E-01	7.16E-01	7.62E-01	2.06E-01	1.07E-01
	Leucine	R	0.35	0.34	0.45	**0.60**	**0.73**	0.49	0.51	0.52	**0.60**	**0.68**	0.28	0.26	0.34	**0.63**	**0.77**
		p(t)	2.59E-01	2.77E-01	1.44E-01	**3.98E-02**	**7.28E-03**	1.10E-01	9.25E-02	8.00E-02	**4.04E-02**	**1.56E-02**	3.73E-01	4.07E-01	2.81E-01	**2.69E-02**	**3.56E-03**
	Methionine	R	0.42	0.46	0.51	**0.78**	**0.82**	0.56	**0.61**	**0.61**	**0.75**	**0.78**	0.36	0.40	0.41	**0.84**	**0.87**
		p(t)	1.72E-01	1.35E-01	8.76E-02	**2.83.10-3**	**1.11E-03**	5.98E-02	**3.44E-02**	**3.36E-02**	**5.09.10-3**	**2.73E-03**	2.47E-01	2.02E-01	1.82E-01	**5.56.10-4**	**2.42E-04**
	Phenylalanine	R	0.41	0.41	0.52	**0.66**	**0.78**	**0.54**	0.56	**0.60**	**0.67**	**0.74**	0.30	0.33	0.41	**0.73**	**0.86**
		p(t)	1.89E-01	1.89E-01	7.98E-02	**2.03E-02**	**2.64.10-3**	**7.00E-02**	5.58E-02	**3.93E-02**	**1.80E-02**	**5.80.10-3**	3.38E-01	3.02E-01	1.81E-01	**7.05E-03**	**3.01.10-4**

Table S2. Correlations between all the variables and the different ways of expressing the accumulation for red wines. Significant values with p (t) ≤ 0.05 highlighted in bold and checked graphically

			**Accumulation = final - initial (µg/L)**					**Accumulation/ time (µg/L) / days)**					**Accumulation/ O2 no SO2 ((µg/L)/ mg/L)**				
			**Isobutyraldehyde**	**2-methylbutanal**	**3-methylbutanal**	**methional**	**Phenylacetaldehyde**	**Isobutyraldehyde**	**2-methylbutanal**	**3-methylbutanal**	**methional**	**Phenylacetaldehyde**	**Isobutyraldehyde**	**2-methylbutanal**	**3-methylbutanal**	**methional**	**Phenylacetaldehyde**
	Initial Fe	R	0.04	0.10	-0.06	-0.32	-0.37	-0.09	-0.07	-0.16	-0.33	-0.38	0.05	0.05	-0.07	-0.49	-0.46
		p(t)	9.00E-01	7.61E-01	8.56E-01	3.16E-01	2.35E-01	7.79E-01	8.17E-01	6.15E-01	3.00E-01	2.23E-01	8.68E-01	8.79E-01	8.20E-01	1.07E-01	1.30E-01
	TPI	R	0.68	0.63	**0.57**	0.55	**0.60**	**0.72**	**0.70**	0.52	0.52	0.58	**0.67**	**0.67**	**0.62**	**0.64**	**0.65**
		p(t)	1.58E-02	2.87E-02	**5.41E-02**	6.54E-02	**4.00E-02**	**8.89E-03**	**1.15E-02**	8.48E-02	8.13E-02	4.94E-02	**1.67E-02**	**1.65E-02**	**3.27E-02**	**2.52E-02**	**2.22E-02**
	pH	R	**0.61**	**0.60**	**0.64**	**0.88**	**0.87**	**0.74**	**0.77**	**0.71**	**0.84**	**0.86**	0.59	0.56	**0.61**	**0.81**	**0.85**
		p(t)	**3.43E-02**	**3.82E-02**	**2.63E-02**	**1.65E-04**	**2.20E-04**	**5.91E-03**	**3.53E-03**	**9.50E-03**	**6.73E-04**	**3.57E-04**	4.42E-02	6.04E-02	**3.40E-02**	**1.40E-03**	**4.20E-04**
	Time of oxidation	R	**-0.75**	**-0.73**	**-0.85**	**-0.94**	**-0.96**	**-0.88**	**-0.90**	**-0.90**	**-0.92**	**-0.94**	**-0.69**	**-0.66**	**-0.79**	**-0.86**	**-0.93**
		p(t)	**5.10E-03**	**7.09E-03**	**4.73E-04**	**6.82E-06**	**1.33E-06**	**1.35E-04**	**5.35E-05**	**7.91E-05**	**2.81E-05**	**5.77E-06**	**1.27E-02**	**1.96E-02**	**2.15E-03**	**3.30E-04**	**1.24E-05**
	Total consumed O2	R	-0.28	-0.30	-0.51	-0.58	-0.54	-0.40	-0.44	**-0.58**	**-0.58**	-0.56	-0.22	-0.26	-0.43	**-0.59**	-0.55
		p(t)	3.79E-01	3.41E-01	8.99E-02	4.82E-02	6.96E-02	1.93E-01	1.52E-01	**4.63E-02**	**4.71E-02**	6.00E-02	4.99E-01	4.17E-01	1.60E-01	**4.44E-02**	6.67E-02
	O2 no SO2	R	0.46	0.55	0.37	0.42	0.41	0.47	0.55	0.35	0.36	0.36	0.44	0.45	0.32	0.20	0.32
		p(t)	1.29E-01	6.27E-02	2.30E-01	1.74E-01	1.80E-01	1.26E-01	6.27E-02	2.63E-01	2.53E-01	2.50E-01	1.55E-01	1.46E-01	3.13E-01	5.41E-01	3.16E-01
	initial Free SO2	R	0.38	0.41	0.07	-0.33	-0.24	0.18	0.18	-0.18	-0.39	-0.34	0.42	0.41	0.12	-0.44	-0.30
		p(t)	2.17E-01	1.82E-01	8.40E-01	3.02E-01	4.55E-01	5.78E-01	5.79E-01	5.71E-01	2.12E-01	2.84E-01	1.74E-01	1.87E-01	7.09E-01	1.51E-01	3.45E-01
	initial total SO2	R	**-0.59**	**-0.63**	**-0.71**	**-0.74**	**-0.72**	**-0.69**	**-0.74**	**-0.73**	**-0.71**	**-0.70**	-0.53	-0.55	**-0.63**	**-0.63**	**-0.67**
		p(t)	**4.19E-02**	**2.69E-02**	**1.01E-02**	**6.04E-03**	**8.92E-03**	**1.37E-02**	**5.81E-03**	**6.69E-03**	**9.75E-03**	**1.16E-02**	7.37E-02	6.46E-02	**2.87E-02**	**2.94E-02**	**1.79E-02**
	Final total SO2	R	-0.46	-0.33	-0.65	-0.50	-0.50	-0.55	-0.45	**-0.65**	-0.54	-0.55	-0.44	-0.38	**-0.66**	**-0.64**	-0.57
		p(t)	1.30E-01	2.98E-01	2.32E-02	1.01E-01	9.53E-02	6.48E-02	1.45E-01	**2.19E-02**	6.75E-02	6.42E-02	1.54E-01	2.24E-01	**1.95E-02**	**2.35E-02**	5.32E-02
Level of native aldehyde	Isobutyraldehyde	R	**-0.85**	**-0.84**	**-0.61**	-0.36	-0.48	**-0.80**	**-0.80**	-0.45	-0.32	-0.40	**-0.84**	**-0.81**	**-0.63**	-0.28	-0.45
		p(t)	**4.04E-04**	**5.70E-04**	**3.50E-02**	2.47E-01	1.15E-01	**1.91E-03**	**1.74E-03**	1.45E-01	3.14E-01	1.99E-01	**6.03E-04**	**1.25E-03**	**2.68E-02**	3.84E-01	1.46E-01
	2-methylbutanal	R	-0.90	-0.91	-0.64	-0.35	-0.34	-0.77	-0.77	-0.44	-0.27	-0.28	-0.93	-0.92	-0.70	-0.26	-0.29
		p(t)	7.07E-05	3.29E-05	2.43E-02	2.69E-01	2.85E-01	3.16E-03	3.17E-03	1.53E-01	4.01E-01	3.85E-01	1.48E-05	1.67E-05	1.09E-02	4.15E-01	3.56E-01
	3-methylbutanal	R	**-0.93**	**-0.91**	**-0.83**	-0.53	-0.50	**-0.87**	**-0.85**	-0.67	-0.48	-0.47	**-0.94**	**-0.94**	**-0.87**	-0.51	-0.49
		p(t)	**9.19E-06**	**3.12E-05**	**7.71E-04**	7.92E-02	9.99E-02	**2.16E-04**	**4.78E-04**	1.66E-02	1.17E-01	1.23E-01	**7.39E-06**	**6.77E-06**	**2.23E-04**	8.86E-02	1.10E-01
	Methional	R	-0.39	-0.48	-0.38	-0.53	**-0.60**	-0.49	**-0.60**	-0.42	-0.50	-0.55	-0.32	-0.36	-0.28	-0.38	-0.55
		p(t)	2.10E-01	1.16E-01	2.25E-01	7.39E-02	**3.78E-02**	1.07E-01	**3.96E-02**	1.71E-01	9.70E-02	6.52E-02	3.13E-01	2.56E-01	3.72E-01	2.24E-01	6.61E-02
	Phenylacetaldehyde	R	-0.18	-0.26	-0.29	-0.49	-0.55	-0.32	-0.42	-0.39	-0.48	-0.52	-0.10	-0.15	-0.18	-0.41	-0.52
		p(t)	5.75E-01	4.22E-01	3.56E-01	1.06E-01	6.59E-02	3.10E-01	1.72E-01	2.16E-01	1.13E-01	8.54E-02	7.65E-01	6.35E-01	5.68E-01	1.82E-01	8.03E-02
Level of native aminoacids	Valine	R	-0.22	-0.20	-0.37	-0.47	**-0.62**	-0.36	-0.38	-0.41	-0.46	-0.56	-0.15	-0.18	-0.32	-0.57	**-0.67**
		p(t)	4.87E-01	5.35E-01	2.33E-01	1.22E-01	**3.21E-02**	2.53E-01	2.22E-01	1.84E-01	1.30E-01	5.73E-02	6.33E-01	5.83E-01	3.09E-01	5.50E-02	**1.66E-02**
	Isoleucine	R	-0.20	-0.20	-0.30	-0.34	-0.53	-0.31	-0.35	-0.31	-0.33	-0.45	-0.13	-0.16	-0.24	-0.40	-0.57
		p(t)	5.25E-01	5.29E-01	3.38E-01	2.78E-01	7.40E-02	3.22E-01	2.60E-01	3.30E-01	2.95E-01	1.40E-01	6.96E-01	6.26E-01	4.53E-01	1.97E-01	5.20E-02
	Leucine	R	-0.34	-0.27	-0.51	-0.57	**-0.75**	-0.51	-0.49	-0.57	**-0.59**	**-0.70**	-0.26	-0.20	-0.44	-0.58	**-0.76**

		p(t)	2.72E-01	3.97E-01	9.27E-02	5.38E-02	**5.11E-03**	9.37E-02	1.04E-01	5.47E-02	**4.24E-02**	**1.07E-02**	4.06E-01	5.30E-01	1.57E-01	5.00E-02	**3.76E-03**
	Methionine	R	-0.46	-0.43	**-0.64**	**-0.87**	**-0.92**	-0.64	**-0.66**	**-0.73**	**-0.84**	**-0.89**	-0.40	-0.40	**-0.58**	**-0.90**	**-0.95**
		p(t)	1.35E-01	1.58E-01	**2.54E-02**	**2.60E-04**	**2.33E-05**	2.60E-02	**1.86E-02**	**6.63E-03**	**5.87E-04**	**9.03E-05**	2.01E-01	2.01E-01	**4.61E-02**	**6.18E-05**	**3.00E-06**
	Phenylalanine	R	**-0.59**	-0.55	**-0.72**	**-0.86**	**-0.95**	**-0.74**	**-0.76**	**-0.77**	**-0.83**	**-0.90**	-0.53	-0.51	**-0.68**	**-0.87**	**-0.96**
		p(t)	**4.40E-02**	6.30E-02	**7.80E-03**	**3.93E-04**	**3.55E-06**	**5.70E-03**	**4.43E-03**	**3.24E-03**	**8.82E-04**	**6.53E-05**	7.77E-02	9.21E-02	**1.59E-02**	**2.57E-04**	**5.66E-07**
Aminoacid increse	Valine	R	0.22	0.20	0.37	0.47	**0.62**	0.36	0.38	0.41	0.46	0.56	0.15	0.18	0.32	0.57	**0.67**
		p(t)	4.87E-01	5.35E-01	2.33E-01	1.22E-01	**3.21E-02**	2.53E-01	2.22E-01	1.84E-01	1.30E-01	5.73E-02	6.33E-01	5.83E-01	3.09E-01	5.50E-02	**1.66E-02**
	Isoleucine	R	0.20	0.20	0.30	0.34	0.53	0.31	0.35	0.31	0.33	0.45	0.13	0.16	0.24	0.40	**0.57**
		p(t)	5.25E-01	5.29E-01	3.38E-01	2.78E-01	7.40E-02	3.22E-01	2.60E-01	3.30E-01	2.95E-01	1.40E-01	6.96E-01	6.26E-01	4.53E-01	1.97E-01	**5.20E-02**
	Leucine	R	0.34	0.27	0.51	0.57	**0.75**	0.51	0.49	0.57	**0.59**	**0.70**	0.26	0.20	0.44	0.58	**0.76**
		p(t)	2.72E-01	3.97E-01	9.27E-02	5.38E-02	**5.11E-03**	9.37E-02	1.04E-01	5.47E-02	**4.24E-02**	**1.07E-02**	4.06E-01	5.30E-01	1.57E-01	5.00E-02	**3.76E-03**
	Methionine	R	0.46	0.43	**0.64**	**0.87**	**0.92**	**0.64**	**0.66**	**0.73**	**0.84**	**0.89**	0.40	0.40	**0.58**	**0.90**	**0.95**
		p(t)	1.35E-01	1.58E-01	**2.54E-02**	**2.60E-04**	**2.33E-05**	**2.60E-02**	**1.86E-02**	**6.63E-03**	**5.87E-04**	**9.03E-05**	2.01E-01	2.01E-01	**4.61E-02**	**6.18E-05**	**3.00E-06**
	Phenylalanine	R	**0.59**	0.55	**0.72**	**0.86**	**0.95**	**0.74**	**0.76**	**0.77**	**0.83**	**0.90**	0.53	0.51	**0.68**	**0.87**	**0.96**
		p(t)	**4.40E-02**	6.30E-02	**7.80E-03**	**3.93E-04**	**3.55E-06**	**5.70E-03**	**4.43E-03**	**3.24E-03**	**8.82E-04**	**6.53E-05**	7.77E-02	9.21E-02	**1.59E-02**	**2.57E-04**	**5.66E-07**
